# Surveillance for the prevention of chronic diseases through information association

**DOI:** 10.1186/1755-8794-7-7

**Published:** 2014-01-30

**Authors:** Juliana Tarossi Pollettini, José Augusto Baranauskas, Evandro Seron Ruiz, Maria da Graça Pimentel, Alessandra Alaniz Macedo

**Affiliations:** 1 Department of Computer Science and Mathematics - FFCLRP - University of São Paulo (USP), Ribeirão Preto-SP, Brazil; 2 Department of Computer Science - ICMC - University of São Paulo, São Carlos-SP, Brazil

**Keywords:** Biomedical informatics, Retrieval and application of biomedical knowledge and information, Medical records and scientific papers, Ontology

## Abstract

**Background:**

Research on Genomic medicine has suggested that the exposure of patients to early life risk factors may induce the development of chronic diseases in adulthood, as the presence of premature risk factors can influence gene expression. The large number of scientific papers published in this research area makes it difficult for the healthcare professional to keep up with individual results and to establish association between them. Therefore, in our work we aim at building a computational system that will offer an innovative approach that alerts health professionals about human development problems such as cardiovascular disease, obesity and type 2 diabetes.

**Methods:**

We built a computational system called Chronic Illness Surveillance System (CISS), which retrieves scientific studies that establish associations (conceptual relationships) between chronic diseases (cardiovascular diseases, diabetes and obesity) and the risk factors described on clinical records. To evaluate our approach, we submitted ten queries to CISS as well as to three other search engines (Google™, Google Scholar™ and Pubmed®;) — the queries were composed of terms and expressions from a list of risk factors provided by specialists.

**Results:**

CISS retrieved a higher number of *closely related* (+) and *somewhat related* (+/-) documents, and a smaller number of *unrelated* (-) and *almost unrelated* (-/+) documents, in comparison with the three other systems. The results from the Friedman’s test carried out with the post-hoc Holm procedure (95% confidence) for our system (control) versus the results for the three other engines indicate that our system had the best performance in three of the categories (+), (-) and (+/-). This is an important result, since these are the most relevant categories for our users.

**Conclusion:**

Our system should be able to assist researchers and health professionals in finding out relationships between potential risk factors and chronic diseases in scientific papers.

## Background

Worldwide chronic diseases are serious health problems and are considered the main cause of mortality among men and women, since they correspond to 60% of all deaths — as observed by the World Health Organization [1]. These illnesses have multifactorial etiologies caused by the interaction of several common factors which include genes, nutrition and lifestyle [2]. An unhealthy diet, physical inactivity and tobacco use are the major risk factors that contribute for the appearance of these disorders. At least 80% of all heart diseases, stroke and type 2 diabetes cases could be prevented if these major risks were eliminated — as also observed by the World Health Organization [3,4].

Genomic medicine has suggested that the exposure to risk factors during early life (at conception, and/or during fetal life, infancy and early childhood) may influence gene expression and consequently induce the development of chronic diseases in adulthood [5]. The presence of premature risk factors can induce variations on several gene expression processes. Since gene interaction and environmental factors play a significant role in complex diseases, it is possible to imply a relationship between early exposure to risk factors and chronic diseases in adults.

In the 90’s, Baker made discoveries that indicated a need for a more thorough study on epigenetics in an attempt to prevent diseases of high prevalence, such as cardiovascular diseases, diabetes and obesity [6,7]. The amount of scientific literature available on health subjects that include research on epigenetics increases every year. For instance, the PubMed repository, a free information repository developed and maintained by the U.S. National Center for Biotechnology Information (NCBI), currently comprises over twenty million citations from biomedical literature from MEDLINE®;. In spite of the positive effects of large amounts of scientific information, there are also some negative aspects. The vast amount of scientific information burdens health care professionals interested in keeping updated, as searches for accurate information are complex and time consuming. As some computational techniques might support the management of large biomedical information repositories and the discovery of knowledge, such techniques and methodologies should be used to retrieve and manage biomedical knowledge, like the premature risk factors for chronic diseases registered in clinical records.

According to Butte [8], public health improvements through the effective transformation of biomedical research results has been considered an important domain of medical informatics — Translational Bioinformatics. The American Medical Informatics Association (AMIA) defines Translational Bioinformatics as “... the development of storage, analytic, and interpretive methods to optimize the transformation of increasingly voluminous biomedical data, and genomic data, into proactive, predictive, preventive, and participatory health” [9].

This work presents a surveillance system that aims to provide health professionals with information regarding the environmental exposures during early life that induce changes in the development which, in turn, pose a long-term impact on later health and disease risk. For example, the association between low-birth weight and prevalent coronary heart disease has been studied. In this case, the people who are at an increased risk of coronary heart disease are those who were small at birth because they failed to grow (usually fetuses with limited nutrients and oxygen) rather than those who were small because they were born early [5]. As another example, health professionals should be aware that, in cases where overweight pregnant mothers expose their fetuses to an imbalanced nutrient supply with excessive amounts of sugar there is a tendency to prenatal undernutrition. Given that escaping from this diet promotes the growth of children more readily than the growth in utero, prenatal undernutrition with retarded growth is followed by improved postnatal nutrition with accelerated growth [10]. Other human development problems associated with environmental exposure are type 2 diabetes and metabolic disturbances, osteoporosis, chronic obstructive lung disease, some forms of cancer and some mental illnesses [11]. In order to favor good life conditions in adulthood, it is necessary to promote information sharing and alert healthcare professionals.

Our computational system, called Chronic Illness Surveillance System (CISS), retrieves scientific papers that relate chronic diseases to risk factors found in the patients’ clinical records. By using our approach, healthcare professionals should be able to create a clinical routine with families and set up the best possible growing conditions.

## Methods

In this section we first present the design and implementation of CISS, followed by the design of the evaluation we report.

### CISS: design and implementation

Several linguistic approaches have been used to process biomedical texts. Due to language complexity, text mining systems usually focus on just one of the following linguistic structure features: words, phrases, semantic concepts or semantic relations. Concept recognition is usually supported by thesauri and/or ontologies.

In order to exploit the scientific results from genomics and bioinformatics and thus contribute to public health, we built the Chronic Illness Surveillance System (CISS), a computational system which was created following systematic methods and methodology. By processing biomedical information, CISS alerts health professionals about risk factors on three chronic diseases: cardiovascular diseases, diabetes mellitus and obesity. This system can be considered a surveillance service because it serves as an early warning system that monitors the epidemiology of a condition to guide health policies and strategies.

**Figure 1:**
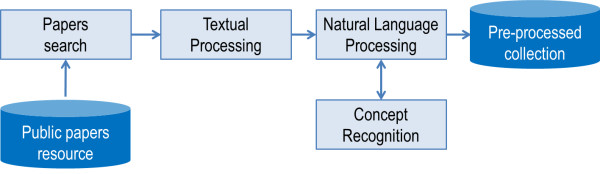
***Use Case 1*– collecting and updating the collection of papers.**

**Figure 2:**
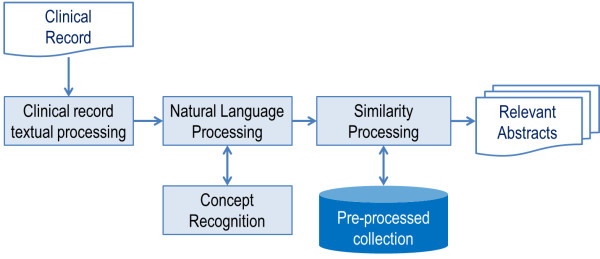
***Use Case 2*– retrieving relevant papers for a clinical record.**

First, CISS prepares and updates a collection of scientific papers in the domain of genetic and epigenetic risk factors for chronic diseases. It does so on a regular basis. Next, the system processes textual information from the papers and stores metadata from this manipulation. Finally, the pre-processed collection is used to retrieve relevant papers according to a clinical record, which was also textually processed. Figure 1 and Figure 2 illustrate these two main use cases: *Use Case 1* – preparation and update of collection of papers, and *Use Case 2* – retrieval of papers that are relevant for a clinical record.

To collect and update scientific papers, which we define as *Use Case 1*, CISS processes textual information from papers, identifying simple and complex terms. These terms are then compared with medical and biomedical concepts from ontologies or thesauri. The recognized terms are statically weighed and stored into a database.

**Figure 3:**
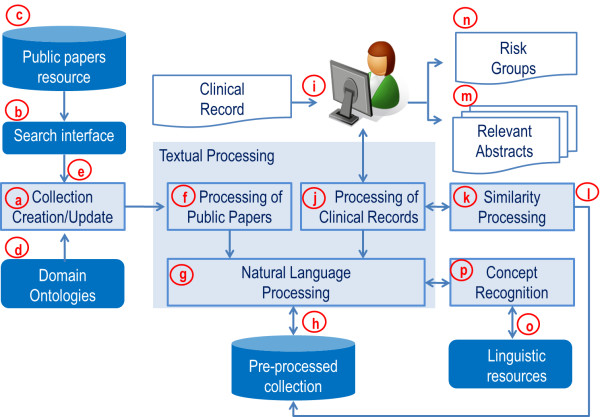
**CISS’s infrastructure (processes and storage).a)** module for Collection Creation/Update; **b)** search interface; **c)** interactions with PubMed; **d)** Ontology concepts on the genetic and epigenetic risk factors domain; **e)** a collection of papers is retrieved from the public repository; **f-g)** textual processing of scientific papers; **h)** local database with a collection of pre-processed scientific papers to support retrieval tasks; **i)** user interface to submission of clinical records; **g-j)** textual processing of the clinical record; **k)** similarity processing among clinical records and scientific papers; **l)** similarity module accesses the pre-processed scientific paper collection and papers with the highest degrees of similarity to the clinical records are retrieved; **m)** the selected papers are shown to the health professional in a graphical user interface; **n)** the user interface has also an option to show a list of risk factors associated with the submitted clinical record; **o)** linguistic resources, like UMLS, that support overall textual processing; **p)** concept recognition module.

To retrieve the papers according to clinical records, which we define as *Use Case 2*, it is necessary to process those records in a similar way to *Use Case 1*. First, terms are identified as simple or complex and compared with linguistic dictionaries. Next, queries are composed by using the manipulated terms. Statistical similarity measures are applied to compute the similarities between queries and papers. Papers with the highest degrees of similarity to the clinical records are retrieved. *Use Case 1* and *Use Case 2* represent requirements and processes for the CISS infrastructure illustrated in Figure 3.

Figure 3 presents the system’s architecture with modules and relationships. CISS has a module called *Collection Creation/Update* (Figure 3a) which, using a search interface (Figure 3b), is responsible for interactions with PubMed (Figure 3c) – the module routinely searches and retrieves papers using the interface that exploits the *Entrez Programming Utilities* and the *Biopython project API*. This module is supported by ontology concepts on the genetic and epigenetic risk factors domain (Figure 3d). The queries submitted to the PubMed service carry *Chronic Disease Ontology* (CDO)^1^ terms and also UMLS *(Unified Medical Language System)*^2^ terms. By using these concepts, a collection of papers is retrieved from the public repository (Figure 3e), textually processed (Figure 3f-g) and then saved for future use in a local database (Figure 3h). This process (Figure 3a-h) is repeated routinely so that new papers included in the public collection are textually processed and the corresponding results are stored into the local database. The main goal here is to compose a collection of pre-processed scientific papers to support retrieval tasks (Figure 3h).

To increase the specificity of terms, as well as to correlate English/Portuguese concepts, the *Concept Recognition* module (Figure 3p) searches concepts from the UMLS, an example of linguistic resource (Figure 3o). The use of UMLS was essential to this work because: (i) it supports the composition of the query executed by the *Collection Creation/Update* module (Figure 3a); (ii) it helps to decrease the number of terms considered by similarity processing among documents (Figure 3k); (iii) it helps to increase the specificity of terms manipulated; and (iv) it correlates terms between English and Portuguese.

In order to retrieve papers related to a clinical record (Figure 3i) submitted by a health professional, CISS first processes the clinical record (Figure 3g and Figure 3j) and then calls a module responsible for processing the similarity among documents (Figure 3k). This module in turn accesses (Figure 3l) the pre-processed version of scientific paper collection (Figure 3h) and papers with the highest degrees of similarity to the clinical records are retrieved. The selected papers are then shown to the health professional in a graphical user interface (Figure 3m). The user interface has also an option to show a list of risk factors (Figure 3n) which are associated with the clinical record submitted by the health professional.

The overall textual processing, supported by linguistic resources (Figure 3o), includes the removal of stopwords, *n*-grams processing, concept recognition (Figure 3p) and the computation 3f weights for the concepts. Stopword removal for the collection of papers is based on the English stopword list from Snowball [12]. The *n*-grams processing uses the Python package NLTK — an open source Python set of modules, linguistic data and documentation for research and development in natural language processing and text analytics [13].

CISS uses the strategy of weighed index terms *tf–idf*, computing frequencies and inverse document frequencies for the concepts contained within the documents to calculate the weight of the concepts of the documents. The processing of clinical records is similar to the processing of documents from the collection of scientific papers. The difference between the two is that, for the clinical records in Portuguese, the removal of stopwords is accomplished by using the Portuguese stopword list from the Snowball project [14]. The completion of the processing of clinical records also differs from the previous one in that, after the *n*-gram processing and the identification of the health concepts associated with the *n*-grams, an array containing the remaining concepts is built and submitted to the process in charge of computing similarities. This process identifies the relationships between clinical records and papers from the collection.

The system uses the *Vector Space Model* to process the similarities among documents. It also creates arrays of terms for each document from the collection using the weights which were calculated during textual processing and stored in the database. These arrays compose a matrix called the *Weight Matrix*.

The *Weight Matrix* corresponds to a vector space in which each document is represented by a vector and each dimension of the space is composed of a concept from a set of classifying terms. The distance between the documents consists in the distance between the vectors scattered in the vector space. Therefore, by using the *Weight Matrix*, it is possible to calculate the cosine of the angle between the vectors of terms, which are representative of the documents, to define the distance between them. The greater the value of the cosine between two vectors, the greater the similarity between them. Thus, the similarity between each document from this *Weight Matrix* and the clinical records is calculated. Results are retrieved in descending order of similarity, i.e., from the most similar to the least similar until a required threshold of similarity, being 0<*threshold*<1, for example, *threshold*=0.015.

### CISS: design of the evaluation

Primary healthcare professionals have direct contact with patients and their families on a regular basis. These professionals can be considered the first and most up-to-date sources of information on the evolution of health patterns of Brazilian families. Therefore, it is critical that primary healthcare professionals obtain useful information about the processes associated with the evolution of family health, which can then be used to support preventive healthcare measures and improve the well-being of individuals and of the society as a whole.

In the field of pediatric care, for example, preventive measures are a vital piece of the process for ensuring quality in child development. Primary healthcare professionals should be able to readily identify children who are at risk and prescribe the necessary intervention to minimize healthcare problems. These professionals should also be able to identify factors that help improve healthcare. The application of computer-aided technologies to mine data and associate healthcare information helps to improve healthcare practices and procedures; for example, these technologies can help to identify children with risk of developing chronic diseases.

Some researchers from the Children’s Institute (ICr) [15] from the Faculty of Medicine at the University of São Paulo, Brazil, support a multiprofessional healthcare group in a community teaching medical center [16] that offers primary healthcare services to approximately two thousand children. A group of researchers from the teaching medical center who specialize in developmental origins of health and disease prepared a set of the most common expressions, terms and values found in the patients’ medical reports at the center. The terms, expressions and values that implied potential risk factors were combined into ten queries in an attempt to evaluate the viability of CISS. We simulated the following scenario: “researchers want to find out relationships between potential risk factors and chronic diseases”.

The ten queries were initially formulated in Portuguese because CISS was built as a preliminary validation of our approach to analyze clinical records in a way that was very transparent to the healthcare professional, and Brazilian physicians are Brazilian Portuguese native speakers. For example, in a fictitious scenario, a clinical record by a health professional in Portuguese was: *“subnutrição materna associada a stress e baixo peso ao nascer” (maternal undernutrition associated with stress and low birth weight)*. By using CISS, it was possible to retrieve some citations from PubMed closely related to the domain of interest (risk factors for chronic diseases). We also evaluated the system’s capability of cross-language information retrieval, as we ran another experiment using five queries in English that corresponded to the first five queries of the ten queries in Portuguese.

The queries were submitted to CISS and to other three search engines, namely Google™, Google Scholar™ and Pubmed®;. The results were collected from 01/24/2013 to 01/28/2013 (Portuguese queries) and from 11/01/2013 to 11/15/2013 (English queries). The first ten documents retrieved from each search engine, including CISS, in both Portuguese and English experiments, were classified into four categories: *closely related* to the specific domain (+), *somewhat related* (+/-), *almost unrelated* to the domain (-/+), and *unrelated* to the specific domain (-).

The criteria adopted to classify results into four categories were: 

• Closely related (+): scientific results (preferably papers) that relate the analyzed query and risk factors for chronic diseases, notably epigenetic mechanisms.

• Somewhat related (+/-): scientific results concerning the query and citing risk for chronic diseases and/or epigenetic mechanisms, but that do not relate them.

• Almost unrelated (-/+): results that comprise only the query, but no risk factors for chronic diseases (e.g. epigenetic mechanisms) or vice versa (in a scientific mode or at least official communication, such as guides from the Health Department), or unscientific results that relate the query to risk factors for chronic diseases.

• Unrelated to the specific domain (-): informal results or results unrelated to the query, or to risk factors for chronic diseases, or to epigenetics.

## Results and Discussion

Queries submitted in Portuguese to PubMed did not return any results for the searches performed. Therefore, a hybrid approach was used to enable comparisons with PubMed, i.e. queries were first translated from Portuguese into English via Google Translator and then submitted to PubMed. This approach, however, was not considered a substitute to CISS’s processes because automatic translations are, up to this moment, not perfect. An imperfect translation could introduce errors to the retrieval process and so we ran experiments using the English queries. These are presented next.

A total of 242 documents from the first ten results from each system were classified, and varying amounts of documents retrieved for each of the four categories (+), (+-), (-+), and (-) were obtained: 

• Google retrieved 79 documents (7, 7, 32 and 33)

• Google Scholar retrieved 69 documents (1, 4, 42 and 22)

• PubMed retrieved 40 documents (10, 4, 11 and 15)

• CISS retrieved 57 documents (32, 12, 10 and 0)

Amongst the above, CISS retrieved a higher number of closely related (+) and somewhat related (+/-) documents, and a smaller number of unrelated (-) and almost unrelated (-/+) documents in comparison with the three other systems.

A detailed comparison is presented in Table 1, which illustrates the results from each of the ten queries (a) to (j). Table 1 also shows that CISS retrieved a higher number of papers related to risk factors for chronic diseases. Considering the five queries in English, the first ten results from each search engine, according to Table 2, were: 

• Google presented 42 results (5, 1, 15 and 21)

• Google Scholar presented 34 results (6, 3, 18 and 7)

• PubMed presented 11 results (0, 4, 6 and 1)

• CISS presented 12 results (2, 6, 4 and 0)

**Table 1:** Number of documents retrieved by CISS and the three other search engines, based on ten queries in Portuguese composed by terms and expressions from a list of risk factors provided by specialists

	**Google**				**Google scholar**				**PubMed**				**CISS**			
	**+**	**+/-**	**-/+**	**-**	**+**	**+/-**	**-/+**	**-**	**+**	**+/-**	**-/+**	**-**	**+**	**+/-**	**-/+**	**-**
a	0	2	6	2	0	2	3	5	0	0	0	0	1	0	0	0
b	0	0	0	0	0	1	6	0	0	0	0	0	3	4	2	0
c	2	0	4	4	0	0	7	3	0	2	4	4	0	0	0	0
d	0	0	2	8	0	0	5	5	0	0	0	0	0	0	2	0
e	3	0	6	1	0	0	0	0	3	2	2	3	6	2	2	0
f	1	1	5	3	1	0	9	0	0	0	0	0	0	0	0	0
g	0	0	0	0	0	0	0	0	7	0	3	0	6	0	0	0
h	0	1	3	6	0	0	3	7	0	0	0	0	8	1	1	0
i	0	0	2	7	0	0	1	1	0	0	0	0	6	2	2	0
j	1	3	4	2	0	1	8	1	0	0	2	8	2	3	1	0

Translated Queries (rows). **(a)** Gestational background: *Did she receive folic acid and iron? When did she start taking each of them and for how long has she been taking them? Has she received other drugs during this pregnancy?***(b)** Gestational background: *Disorders during pregnancy, such as hypertension, HDP, preeclampsia, eclampsia, heart disease, diabetes mellitus, gestational diabetes, anemia, bleeding (if positive, in which trimester), maternal infections during pregnancy such as toxoplasmosis, CMV, AIDS, syphilis, UTI, Leucorrhoea, Streptococcus, reported stress and its cause.***(c)** Maternal background: *age at menarche.***(d)** Child’s everyday life: *hours of sleep per day, sleep disorders, playing routine.***(e)** Newborn background: *low-weight TNB, low-weight PTNB, metabolic disease, nutritional disease.***(f)** Environmental conditions: *housing situation in regards to water and sewer systems, and physical space*. **(g)** Diagnostics, medical managements, treatment and referrals: *eutrophia, overweight or obesity, high BMI, body mass excess, adiposity excess, or malnutrition sign, dystrophy, iron deficiency, anemia, rickets.***(h)** Family background: *history of diseases and habits of the nuclear family (father, mother, brothers, maternal grandparents, paternal grandparents and others), consanguinity between parents.***(i)** Familiogram: *presence of several individuals with chronic diseases, sequelae from chronic disease, environmental risk for chronic disease, teenage parents, loss of family members by chronic disease.***(j)** Family risks and protection related to health: *mother or relatives with psychiatric problems or special needs.*

**Table 2:** Number of documents retrieved by CISS and three other search engines, based on five queries composed in English by terms and expressions from a list of risk factors provided by specialists

	**Google**				**Google scholar**				**PubMed**				**CISS**			
	**+**	**+/-**	**-/+**	**-**	**+**	**+/-**	**-/+**	**-**	**+**	**+/-**	**-/+**	-	**+**	**+/-**	**-/+**	**-**
a	0	0	1	9	0	1	6	3	0	0	0	0	1	0	0	0
b	0	0	0	2	1	2	0	0	0	0	0	0	0	1	0	0
c	4	1	2	3	3	0	3	4	0	4	5	1	0	4	4	0
d	1	0	5	4	2	0	8	0	0	0	1	0	0	1	0	0
e	0	0	7	3	0	0	1	0	0	0	0	0	1	0	0	0

Original Queries (rows): **(a)** Gestational background: *Did she receive folic acid and iron? When did she start taking each of them and how long has she been taking them? Has she received other drugs in this pregnancy?***(b)** Gestational background: *disorders during pregnancy such as hypertension, HDP, preeclampsia, eclampsia, heart disease, diabetes mellitus, gestational diabetes, anemia, bleeding (if so, in which trimester), maternal infections during pregnancy such as toxoplasmosis, CMV, AIDS, syphilis, UTI, Leucorrhoea, Streptococcus, reported stress and its cause.***(c)** Maternal background: *age at menarche.***(d)** Child’s everyday life: *sleep (hours of sleep per day, sleep disorders), playing routine.***(e)** Newborn background: *low-weight TNB, low-weight PTNB, metabolic disease, nutritional disease.*

Google Scholar and CISS obtained a higher number of closely related (+) and somewhat related (+/-) documents. In comparison with CISS, Google Scholar retrieved a higher percentage (25 out of 34, 74%) of unrelated (-) and almost unrelated (-/+) documents than CISS (4 out of 12, 33%).

Table 3 shows that CISS retrieved an average of 3.2 papers that are closely related to the evaluated queries (our domain of interest), whereas the average retrieved by Google, Google Scholar and PubMed vary from 0.7 to 1. Yet another positive point is that CISS retrieved an average of 0.0 papers unrelated to the specific domain, whereas the others retrieved an average of more than 1.5 papers. Given that researchers or health professionals are looking for potential risk factors and chronic diseases closely related to their clinical cases, it is important to reduce the number of unrelated results.

**Table 3:** Average number of documents retrieved by CISS and the three other approaches in Portuguese

**Search engine**	**+**^**§**^	**+/-**^**§**^	**-/+**^**§**^	**-**^**§**^	**Mean**^**‡**^	**Median**^**†**^
Google	0.7	0.7	3.2	3.3	157529.3	4810.0
Google scholar	0.1	0.4	4.2	2.2	2180.6	218.5
PubMed	1.0	0.4	1.1	1.5	1789.8	0.0
CISS	3.2	1.2	1.0	0.0	7.9	6.0

^§^Columns relative to the 10 first results for each query at each engine.

^‡^Mean of all results for each query.

^†^Median of all results for each query.

Our qualitative evaluation also verified that the relevant documents retrieved by CISS presented a higher accuracy rate when compared to the other search engines, as is summarized in Table 4. The accuracy rate was calculated by considering, as relevant documents, the papers closely related and somewhat related to the queries.

**Table 4:** Accuracy of the results retrieved by CISS and the 3 other approaches in Portuguese

**Search Engine**	**Google**	**Google scholar**	**PubMed**	**CISS**
% queries at whichthe search engineachieved the bestresults ((+) and (+/-))	15%	15%	10%	60%
Accuracy (precision)	0.14	0.05	0.14	0.61

The average numbers of retrieved documents by category considering the English queries obtained almost the same results as the Portuguese queries (see Table 5). Except for closely related documents (+), CISS performed worse than Google Scholar. However, CISS showed a better accuracy and a higher percentage of queries at which CISS achieved the best results for closely related and somewhat related categories, according to our qualitative evaluation (see Table 6).

**Table 5:** Average number of documents retrieved by CISS and the 3 other approaches in English

**Search engine**	**+**^**§**^	**+/-**^**§**^	**-/+**^**§**^	**-**^**§**^	**Mean**^**‡**^	**Median**^**†**^
Google	1.0	0.2	3.0	4.2	14028687	255000
Google scholar	1.2	0.6	3.6	1.4	12058.8	1190
PubMed	0.0	0.8	1.2	0.2	18.0	0.0
CISS	0.4	1.2	0.8	0.0	2.4	1.0

^§^Columns relative to the 10 first results for each query at each engine.

^‡^Mean of all results for each query.

^†^Median of all results for each query.

**Table 6:** Accuracy of the results retrieved by CISS and the 3 other approaches in English

	**Google**	**Google scholar**	**PubMed**	**CISS**
% queries at whichthe search engineachieved the bestresults ((+) and (+/-))	20%	40%	0%	40%
Accuracy (precision)	0.12	0.32	0.10	0.90

In Table 1, it is also possible to observe that CISS achieved the best results for six of the queries, whereas the other search engines achieved the best result only for one query each. For query (g), there was a tie at one between Google and Google Scholar. Normally, CISS achieved the best performance when manipulating queries composed of specific medical terms. We estimate that this is because we use UMLS to manipulate abbreviations and specific terms. For query (d), none of the engines provided good results; for (c) and (f), Google was the best. This analysis gives the proportion of queries for which each search engine achieved the best results (see Tables 4 and 6).

We used the Friedman Test [17,18] to compare the four experimental situations: Google, Google Scholar, PubMed and CISS in Portuguese and English: the results are summarized in Tables 7 and 8, respectively. Tables 7 and 8 show the results of the Friedman’s Test using the post-hoc Holm procedure (95% confidence) for CISS (control) versus the three other engines, for each category (+), (+/-), (-/+), and (-). In the table, ▵ (▿) indicates that CISS was better (worse) than the corresponding engine, and ▴ (▾) indicates that CISS was significantly better (significantly worse) than the corresponding engine.

**Table 7:** Comparison considering Friedman’s Test for CISS versus all (in Portuguese)

**CISS**	**Google**	**Google scholar**	**PubMed**
+	△	△	△
+/-	△	△	△
-/+	△	△	▿
-	▴	△	△

**Table 8:** Comparison considering Friedman’s Test for CISS versus All (in English)

**CISS**	**Google**	**Google scholar**	**PubMed**
+	^‡^ ∘	▿	△
+/-	△	△	△
-/+	△	△	△
-	▴	△	△

^‡^ ∘ means *No difference whatsoever*.

In Table 7, it is possible to notice that CISS was better than the three other search engines in almost all experimental conditions, except for PubMed (-/+). We are able to verify the significance of the category (-) with a 95% confidence interval.

It is important to observe that CISS had the best performance in categories (+), (+/-) and (-).This is an important result since categories (+) and (+/-) are the categories of interest for our users, and category (-), with zero results, prevents our users from wasting time.

In Table 8, it is possible to notice that CISS had the best performance in categories (+/-), (-/+) and (-). In English, Google Scholar achieved the best performance only for category (+) with a 95% confidence interval. Our results indicate that CISS was efficient at categories (-/+) and (-), thus preventing our users from wasting time.

CISS can be adapted to deal with different collections of scientific papers, different linguistic resources, as well as different kinds of structured documents. The surveillance system was initially instantiated to deal with clinical records in Portuguese and abstracts in English from PubMed. However, the proposed system (CISS) could recognize and relate concepts from any language supported by UMLS. Consequently, it could retrieve and relate scientific papers and clinical records in any language from the exploited linguistic resource.

PubMed had a poor performance in both experiments. We believe that this was partially caused because of the extra steps involved in the Portuguese experiment (i.e. translation of terms), but mainly due to PubMed’s limitation in dealing with natural language expressions. As for the experiment in English, only two out of the five queries retrieved results. One of the main problems of the queries which retrieved no results was the use of acronyms: in fact, PubMed returned 21 results for query (e) after the manual removal of the acronyms (i.e. *“Newborn, low-weight TNB, low-weight PTNB, metabolic disease, nutritional disease”* was manually converted to *“Newborn low-weight term newborn low-weight preterm newborn metabolic disease nutritional disease”*). However, given the problem addressed, the manual conversion is not an interesting approach, since (i) it depends on the user’s knowledge and (ii) it could not be applied, for example, to an application analyzing clinical records in a way that was transparent to the health care professional. CISS, on the other hand, could automatically recognize the concepts related to the majority of the acronyms of the evaluated queries.

## Related work

The development of systems to retrieve relevant biomedical documents on a specific topic is a scientific effort that needs contributions mainly from areas that involve machine learning, information retrieval, information extraction and natural language processing. Question Answering (QA) is a kind of information retrieval system that aims to precisely answer natural language questions posed by humans. In the biomedical domain, QA systems usually need to go beyond lexico-syntactic analysis so that it also includes semantic analysis and processing of textual, terminological, and ontological resources. Athenikos & Han [19] presented an extensive survey about biomedical QA that involved architecture, semantic knowledge and future directions, whereas Kolomiyets & Moens [20] depicted QA technological aspects considering information retrieval.

In the same direction of QA, many biomedical IR systems exploit passages, key phrases and aspects retrieval to return more precise results. Yin et al. [21] proposed a method to find passages to cover different aspects of a query in the biomedical domain. They have used Wikipedia and TREC 2006 and 2007 Genomics to demonstrate the effectiveness of their proposal. Also using the same TREC Genomics, Karimi et al. [22] reported the use of an automatic concept recognition task when using passage and full-text retrieval. Si et al. [23] investigated the combination of multiple biomedical resources (query expansion with UMLS, passages, TREC Genomics and passage retrieval) to support *aspect retrieval* in terms of topical relevance and topical novelty. In TREC 2005 Genomics, Lin et al. [24] had already presented an approach for retrieving biomedical documents considering only *key phrases* for query expansion and taking into account Mesh terms and MEDLINE entries.

Patrick & Li [25] proposed extensions to QA systems in order to answer clinical questions: a question processing with semantic knowledge (SNOMED and a clinical medical taxonomy) and a classification model. To better represent queries, Ryu & Choi [26] applied multiple queries instead of an extension considering semantic knowledge. They collected the top ranked documents in retrieved sets to generate aspect queries.

Aljaber et al. [27] used terms of citation contexts and UMLS to improve the MeSH classification of biomedical articles. Considering the same source of information, Ortuño et al. [28] exploited cited references to support the retrieval of related biomedical documents.

Gobeil et al. [29] compared the assignment of Gene Ontology (GO) terms from a MEDLINE abstract with a machine learning algorithm (k-NN). Having evolved across time in regards to the growth of resources, their system showed a better ability when considering the k-NN algorithm to propose, for a given abstract, the GO terms used for curation in GO Annotations.

A noteworthy amount of scientific papers applies techniques for the recognition of concepts, mainly by mapping terms from the texts to UMLS concepts (e.g. [30-33]). Many authors have also experimented Cross Language Information Retrieval (CLIR) using the multilingual UMLS metathesaurus to translate queries from one language to another.

In 2013, BMC Medical Genomics published several selected articles about translational bioinformatics. Kim [34] advocated that better methods and tools are essential for a successful translation. Kim et al. [35] developed a new algorithm and software to combine haplotype clusters for diplotype-based association analysis. Van de Wiel et al. [36] proposed ShrinkHT, a Bayesian method for shrinking multiple parameters in a statistical model where ‘shrinkage’ refers to borrowing information across features. They applied the method High-throughput RNA Interference (RNAi)screening experiments to overcome the small sample size by borrowing information. Kim et al. [37] proposed OMDR (Ordinal Multifactor Dimensionality Reduction) to facilitate gene-gene interaction analysis for ordinal traits.

In 2012, other scientific efforts exploited associations between bioinformatics or genomic information with scientific papers or semantic knowledge from terminological, and ontological resources. For instance, Chen et al. [38] proposed a method to identify CRGs (Chemosensitivity Related Genes) based on GO (Gene Ontology) and PPIN (Protein Interaction Networks). They (i) documented 150 pairs of drug-CCRG (curated CRG) from 492 published papers, (ii) characterized CCRGs from the perspective of GO and PPIN, (iii) prioritized CRGs based on CCRGs’ GO and network characteristics, and lastly, (iv) evaluated the performance of the proposed method, and obtained good results. Their method identified CRGs with expression patterns strongly correlated with drug activity and also identified CRGs where expressions were weakly correlated with drug activity.

Nevertheless, we have not found related research comprising all of the technologies covered by our proposal, nor a work with the same translational bioinformatics goal: the prevention of chronic diseases through alerting healthcare workers about risk factors, by retrieving published scientific papers with knowledge on epigenetic risk factors.

## Conclusions

Genomic medicine has suggested that the exposure to risk factors from conception to adulthood may influence gene expression and consequently induce the development of chronic diseases. Scientific papers with these discoveries indicate that epigenetics must be exploited in an attempt to prevent high prevalence diseases (such as cardiovascular disease, diabetes and obesity). The developed system aims to alert healthcare professionals about human development problems, such as the incidence of chronic diseases. This goal was achieved by automatically identifying scientific papers that relate chronic diseases with risk factors from patients’ clinical records. As a result, it is expected that health professionals, supported by the discovery of information from scientific papers, create a routine that will establish better growth conditions. Our surveillance system (CISS) should be able to assist researchers and health professionals in pediatrics and other areas. These professionals will be able to discover relationships between potential risk factors and chronic diseases by using CISS to look for these potential factors (from clinical records) in scientific papers. Another possible application might be to use CISS as a recommender system running in parallel with a health information system or an electronic patient record, to analyze clinical records in a way that is very transparent to the healthcare professional in attendance. This system could, for example, analyze a clinical record stored in the database and send an alert with a scientific paper evidencing any association with risk factors for chronic diseases that were eventually found. In this case, the retrieved papers should be presented in smaller numbers to be more specific. In the first scenario of research in pediatrics, papers will support scientific investigations and a higher recall might be more interesting to enlarge the research.

According to Butte [8], the effective transformation of results from biomedical research into knowledge that actually improves public health has been considered to be an important domain of informatics and is known as Translational Bioinformatics. Considering that chronic diseases are a serious worldwide health problem, being the leading cause of mortality (60% of all deaths), our effort might be a first step towards allowing scientific results from bioinformatics to reach the public health service.

As an ongoing work, we plan to classify patients into risk groups, as we have already done in another medical scenario [39]. As future work, we intend to extend the Chronic Disease Ontology (CDO) with the knowledge obtained from scientific papers retrieved by CISS on epigenetic mechanisms and epigenetic risk factors for chronic diseases. This knowledge, however, must be validated by experts before they are permanently embedded into the ontology.

## Endnotes

^1^ Verma et al. [2] have developed the Chronic Disease Ontology (CDO) to store, reuse and discover new knowledge from three types of chronic diseases: cardiovascular diseases, type 2 diabetes and obesity. The CDO includes information about relations between genes and mutations, as well as health, nutrition and life history data.

^2^ The Unified Medical Language System (UMLS), maintained by the US National Library of Medicine, is an important source of information that contributes to the processing and management of biomedical documents. The UMLS comprises a metathesaurus, a semantic network and a specialist lexicon. This set of technologies and knowledge sources was designed to be used by a variety of applications, minimizing the problem of different ways into which a concept can be expressed on biomedical information sources [40].

## Competing interests

The authors declare that they have no competing interests.

## Authors’ contributions

This work is the result of JTP master thesis. JTP contributed to: (i) the conception and design of the study, (ii) the acquisition of data, (iii) the design of experiments, (iv) the analysis and interpretation of data, (v) drafting the article and revising it critically, and (vi) the final approval of the submitted version. ESR helped to draft a first manuscript and discussed scientific concepts. JAB and MGP contributed to the analysis of the results and to the preparation of the manuscript. AAM is the advisor of this study. She coordinated each developing phase of the investigation. She also drafted the manuscript, critically revised the article and granted final approval of the version to be submitted. All authors read and approved the final manuscript.

## Pre-publication history

The pre-publication history for this paper can be accessed here:

http://www.biomedcentral.com/1755-8794/7/7/prepub
